# Management and outcomes of patients presenting with sepsis and septic shock to the emergency department during nursing handover: a retrospective cohort study

**DOI:** 10.1186/s12873-018-0155-8

**Published:** 2018-01-18

**Authors:** Sami Alsolamy, Atheer Al-Sabhan, Najla Alassim, Musharaf Sadat, Eman Al Qasim, Hani Tamim, Yaseen M Arabi

**Affiliations:** 1 Emergency Medicine and Intensive Care Department, College of Medicine, King Saud Bin Abdulaziz University for Health Sciences, King Abdulaziz Medical City, Riyadh, Kingdom of Saudi Arabia; 2 Emergency Medicine Department, King Abdulaziz Medical City, Riyadh, Kingdom of Saudi Arabia; 3 Intensive Care Department, King Abdulaziz Medical City, Riyadh, Kingdom of Saudi Arabia; 4 King Saud Bin Abdulaziz University for Health Sciences, King Abdullah International Medical Research Center, Riyadh, Kingdom of Saudi Arabia; 5 Department of Internal Medicine, American University of Beirut- Medical Center, Beirut, Lebanon; 6 College of Medicine, King Saud Bin Abdulaziz University for Health Sciences, Riyadh, Kingdom of Saudi Arabia; 7 Respiratory Services, King Abdulaziz Medical City, Riyadh, Kingdom of Saudi Arabia

**Keywords:** Sepsis, Handover, Nursing

## Abstract

**Background:**

Clinical handover is an important process for the transition of patient-care responsibility to the next healthcare provider, but it may divert the attention of the team away from active patients. This is challenging in the Emergency Department (ED) because of highly dynamic patient conditions and is likely relevant in conditions that requires time-sensitive therapies, such as sepsis. We aimed to examine the management and outcomes of patients presenting with sepsis and septic shock to the ED during nursing handover.

**Methods:**

This retrospective cohort study was conducted at a 115-bed ED and more than 200,000 annual ED visits, within a 900-bed academic tertiary care center. Data on Surviving Sepsis Campaign (SSC) bundle elements and hospital mortality were collected for all ≥14-year-old patients who presented to the ED with a diagnosis of sepsis and septic shock between January 1, 2011 and October 30, 2013. Our primary outcome was time to antibiotics, were other SSC bundle elements and mortality counted as secondary outcomes. Patients were divided into two groups: 1) handover time group, comprising patients who presented an hour before or after the start of handover time (6–8 AM/PM), and 2) non-handover time group, comprising patients who presented over the remaining 20 h.

**Results:**

During the study period, 1330 patients presented with sepsis or septic shock (228, handover time group; 1102, non-handover time group). No significant differences were found between the handover time and non-handover time groups, respectively, in median time to antibiotic administration (100 [interquartile range (IQR) 57–172] vs. 95 [IQR 50–190] minutes; *P* = 0.07), median time to serum lactate result (162 [IQR 108–246] vs. 156 [IQR 180–246] minutes; *P* = 0.33) and median time to obtain blood culture (54 [IQR 36–119] vs. 52 [IQR 28–103] minutes; *P* = 0.52), and hospital mortality rate (29.4% vs. 28.9%; *P* = 0.89).

**Conclusion:**

No significant differences were found in median time of SSC bundle elements or hospital mortality between patients who presented during the handover and non-handover times.

## Background

Handover is an essential process in the Emergency Department (ED), as patient care is provided in a continuum around the clock manner; it follows, staff must work in shifts [1]. Handover is defined as a transition of care, responsibility, and future management or disposition plans to the next healthcare provider [2]. This definition applies to all health care workers, including physicians, nurses, and care assistants.

Nursing handover is a complex process that requires effective transfer of all required patient information in the most time-efficient manner. This process needs good communication skills and time management. Trivial miscommunication may lead to delivery of inaccurate or incomplete data, resulting in delayed care or other adverse effects [3, 4]. Nursing handover time has been recognized as a time where adverse events are more likely to occur [4]. In addition, the quality of handover has a direct effect on the quality of patient care over the following shift [5]. In the ED, this complexity is further increased because new patients, in stable or unstable condition, can arrive at any time, regardless of the handover time. A prospective observational study addressing ED handover problems revealed deficiencies in the handover processes [4]. These deficiencies were mainly noted in communication and disposition of information [4]. In another study that assessed the differences in information retention between various handover styles, the authors concluded that purely verbal handover processes are even more prone to serious data loss [6]. The lack of transfer of some information during the handover process may significantly reduce the overall quality of patient care [4, 7]. Therefore, it is crucial to investigate delays in treating patients arriving at the ED during handover time.

Reviewing the literature, we found ED handover studies that focused on evaluating the quality of care transfer, the need to initiate a standardized tool to aid the process, and on reporting handover-related errors and adverse events. Interestingly, we did not find any studies that investigated the effect of handover time on the patients with time-sensitive disorders who visited the ED during the nursing handover time. Sepsis is an important time-sensitive condition in which delays in providing care, such as delays in antibiotic administration, are associated with adverse outcomes. In particular, delays in antibiotic administration have been shown to be associated with a 7.6% decrease in survival for each hour of delay in antimicrobial administration during the 6 h after the first hour of documented hypotension [8]. In this study, we aimed to evaluate the direct impact of handover time on the management of sepsis. We compared different processes measurements to reflect the quality of sepsis care, including the time to intravenous antibiotic administration, time to serum lactate result and time to obtain blood culture among patients who arrived at the ED during the nursing handover time in comparison to those who arrived at other times. We hypothesized that arrival at the ED during the nursing handover period is associated with delay in management of septic patients, and with worse outcomes.

## Methods

### Study design and setting

The study was performed in a large, urban, tertiary-care ED with an Emergency Medicine Residency Program. The ED is staffed with board-certified emergency medicine physicians, and has 115 beds. The number of annual ED visits range from approximately 200,000 to 214,000 per year at this 900-bed academic tertiary care center. In this study, we used data from the Sepsis Database, collected as part of a quality improvement project conducted by the Intensive Care and Emergency Medicine Departments. The Sepsis Database used the 2008 and 2012 Surviving Sepsis Campaign (SSC) tools.

### Selection of participants

We included all patients aged ≥14 years who were admitted to the ED with a diagnosis of sepsis and septic shock between January 1, 2011 and October 30, 2013. In our institution, nursing shifts are 12-h based in all departments. Nursing handover process is done at the bedside using both verbal and written forms. The form used in our institution is SBAR (Situation, Background, Assessment, Recommendation) [9], which must be filled by the endorsing nurse to the receiving nurse. For operative purposes, sepsis was defined as systemic inflammatory response syndrome with acute organ dysfunction secondary to documented or suspected infection. Septic shock was defined as sepsis with persistent hypotension after fluid resuscitation with at least 20 mL/kg of crystalloid (or equivalent) [10]. Patients were identified as having sepsis or septic shock based on clinical assessments performed by the ED physicians. We divided these patients into two groups: 1) a handover time group that included patients who visited the ED an hour before or an hour after the handover time (6–8 AM and PM), and 2) a non-handover time group that included patients who arrived at the ED over the remaining 20 h. We chose the 2-h duration to study the effect of the handover because nurses typically start to prepare for the handover one hour prior to the handover time, and handover sessions can last almost an hour after their initiation.

### Methods and measurements

We extracted the following data from the Sepsis Database: ED arrival time, source of infection, physical examination findings, laboratory findings, time-to-antibiotic administration (from arrival to ED), time-to-lactate results and time-to-obtaining blood culture, mechanical ventilation requirement, and hospital mortality [10]. The primary study outcome was the time-to-antibiotic administration, time-to-lactate results and time-to-obtaining blood culture. The study was approved by the Institutional Review Board of Ministry of National Guard-Health affairs, and the informed consent requirement was waived.

### Data analysis

Because of the skewed data distribution, we have presented data as median and inter-quartile range for continuous variables, and frequency and proportion for categorical variables. Continuous variables were compared between two groups using the *t*-test, and categorical values were compared using the chi-square test. All data management and analysis was performed with SAS (version 9.1; SAS Institute, Inc., Cary, NC).

## Results

### Baseline characteristics

Over the study period, 1330 patients fulfilled the diagnostic criteria for sepsis and septic shock, and were included in the final analysis: 228 patients in the handover time group and 1102 patients in the non-handover time group.

The presenting characteristics of patients who arrived during the handover time and those who arrived during the non-handover time are presented in Table 1. The predominant sources of infection in both groups were pneumonia and urinary tract infection. Patients presenting with septic shock made up 38.6% of patients in the handover time group and 40.9% of patients in the non-handover time group. The proportion of patients requiring mechanical ventilation was similar in both groups (29%). Lastly, non-handover group did not differ from handover group patients in terms of initial signs and symptoms nor lab results (Table 1).

**Table 1:** The presenting characteristics of patients who arrived during the handover time and those who arrived during the non-handover time

	Handover time	Non-Handover time	*P*-value
All patients	*N* = 228	*N* = 1102	
Source of sepsis, no. (%)			
Pneumonia	103 (45.2)	499 (45.3)	0.98
Urinary tract infection	32 (14)	173 (15.7)	0.53
Acute abdominal infection	15 (6.6)	80 (7.3)	0.72
Soft tissue infection	6 (2.6)	42 (3.8)	0.38
Other infections	84 (36.8)	376 (34.1)	0.43
Signs and Symptoms, no. (%)			
Temperature > 38 °C	57 (25)	281 (25.5)	0.87
Temperature < 36 °C	6 (2.6)	41 (3.7)	0.42
Acutely altered mental status	47 (20.6)	217 (19.7)	0.75
Chills and rigors	2 (0.9)	18 (1.6)	0.39
Heart Rate > 90/min	201 (88.2)	945 (85.8)	0.34
Respiratory Rate > 20/min	197 (86.4)	928 (84.2)	0.40
Hypotension*	75 (32.9)	332 (30.1)	0.41
Hypoxia*	75 (32.9)	332 (30.1)	0.41
Laboratory Findings, no. (%)			
Leukocytosis*	99 (43.4)	452 (41)	0.50
Leukopenia*	14 (6.1)	49 (4.5)	0.27
Increased creatinine*	13 (5.7)	99 (9)	0.10
Thrombocytopenia*	8 (3.5)	33 (3)	0.68
Hyperbilirubinemia*	5 (2.2)	41 (3.7)	0.25
Hyperlactatemia*	100 (43.9)	458 (41.6)	0.52
Coagulopathy*	13 (5.7)	57 (5.2)	0.74

*Hypotension: systolic blood pressure < 90, mean arterial pressure < 65 or systolic blood pressure decrease > 40 mmHg from baseline, *Hypoxia: oxygen requirement to maintain oxygen saturation > 90%, *Leukocytosis: WBC count > 12 Å~ 109/L, *Leucopenia: white blood cell count < 4 Å~ 109/L, *Increased creatinine: creatinine increase > 176.8 mmol/L, *Thrombocytopenia: platelet count < 100 Å~ 109/L, *Hyperbilirubinemia: bilirubin > 34.2 mmol/L, *Hyperlactatemia: lactate > 2 mmol/L, *Coagulopathy: international normalized ratio (INR) > 1

### Processes of care and outcomes

**Fig. 1:**
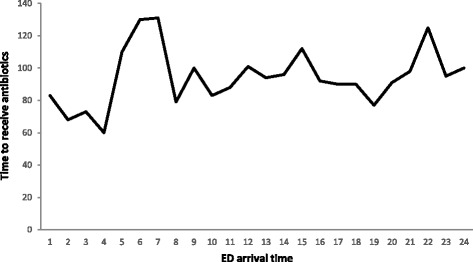
The distribution of median time-to-antibiotic administration by hour of day with the arrival in ED

All patients received antibiotics, and median time-to-antibiotic administration showed a tendency of being longer in the handover time group (100 [IQR 57–172] minutes) as compared to the non-handover time group (95 [IQR 50–190] minutes; *P* = 0.07). The distribution of median time-to-antibiotic administration by hour of day is presented in Fig. 1. The median time-to-lactate results in the handover time group (162 [IQR 108–246] minutes) was not significantly different from that in the non-handover time group (156 [IQR 180–246] minutes; *P* = 0.33). The median time-to-obtaining blood cultures in the handover time group (54 [IQR 36–119] minutes) was not significantly different from that in the non-handover time group (52 [IQR 28–103] minutes; *P* = 0.52). The hospital mortality rate in the handover time group (29.4%) was not significantly different from that in the non-handover time group (28.9%; *P* = 0.89).

## Discussion

Handovers are pivotal junctures and integral process in the continuity of care in every patient’s clinical course. To the best of our knowledge, the idea of handover time as a possible distractor that might delay urgent patient care is not addressed in current literature. We aim to shed light on the duration of handover process as a possible time where patient care is affected. In this study, we evaluated the direct effect of nursing handover process on patient care. We used a sepsis database to compare ED processes and outcomes between patients who arrived at the ED during nursing handover time and those who arrived during non-handover time.

Our results showed a trend of longer time-to-antibiotic administration in handover group, however this was not clinically nor statistically significant. Nonetheless, the clinical value is unconvincing; as 5 min’ difference, might be minor when it comes to antibiotic delivery. It follows, additional studies with another time sensitive assessment tools are needed to address the clinical outcome of this delay, and how to prevent it. We found no significant association between ED nursing handover and time-to- lactate results or time-to-obtaining blood culture, or hospital mortality in patients admitted with a diagnosis of sepsis and septic shock. This could be explained by our ED nurses are vigilant with alerts and our institution was conducting SSC with constant reminders of early management and septic alerts [11]. Consequently, our results may not reflect the situation in institutions with different methods of handover.

A prospective observational study addressing ED handover problems revealed deficiencies in the handover processes [4]. These deficiencies were mainly noted in communication and disposition of information [4]. In another study that assessed the differences in information retention between various handover styles, the authors concluded that purely verbal handover processes are even more prone to serious data loss [6]. In light of that, researchers have been developing new tools to ease the process and grant adequate transfer of information [9]. These tools have been shown to improve nursing handover [12–19].

The main strengths of our study include the numbers of patients included, detailed data collection, the tertiary academic setting with numerous complex and critically ill patients, and standardized data collection using the SSC tools. As a retrospective cohort study, the present study has some important limitations. Foremost, the study aimed to evaluate the direct impact of handover time on time to time-to-antibiotic administration, time-to-lactate results, and time-to-obtaining blood culture, as surrogate indicators of the quality of sepsis and septic shock management. Nonetheless, other important measures in sepsis management were not investigated such as time to effective fluid resuscitation. Additionally, there is inherent variation and subjectivity in the handover process among ED nursing staff might have underpowered our results. Lastly, because of the retrospective nature of this study and the fact that it was conducted in a busy ED, others factors, such as ED overcrowding and boarding patients in ED, could have affected the study results.

## Conclusion

This is one of the first reports of the impact of ED nursing handover on time-sensitive interventions that involve multiple tasks performed by ED nurses. Due to the retrospective nature, patient population with single pathology, and our structured handover process that might have reflected on the results of this study. Future studies are still needed to explore ED functionality during the handover time.

## Abbreviations

ED: Emergency department
IQR: Interquartile range
SAS: Statistical analysis software
SSC: Surviving sepsis campaign
